# Emerging Immunotherapy and Antibody-Derived Therapeutics for the Treatment of Advanced Non-Small-Cell Lung Cancer: A Review

**DOI:** 10.3390/cancers18081291

**Published:** 2026-04-19

**Authors:** Alicia Yunxin Hou, Dina Elantably, Rami Manochakian, Vamsidhar Velcheti, Shenduo Li, Yujie Zhao, Yanyan Lou

**Affiliations:** 1 Department of Internal Medicine, Mayo Clinic, Jacksonville, FL 32224, USA; hou.alicia@mayo.edu; 2 Division of Hematology and Medical Oncology, Department of Internal Medicine, Mayo Clinic, Jacksonville, FL 32066, USA; elantably.dina2@mayo.edu (D.E.); manochakian.rami@mayo.edu (R.M.); velcheti.vamsidhar@mayo.edu (V.V.); li.shenduo@mayo.edu (S.L.); zhao.yujie@mayo.edu (Y.Z.)

**Keywords:** NSCLC, immunotherapy, immune checkpoint inhibitors, monoclonal antibodies, bispecific antibodies, antibody–drug conjugates, cellular therapies, cancer vaccines, adoptive cell therapies, emerging therapies

## Abstract

**Background**: In the last decade, a surge of advancements in and approvals of immunotherapies has revolutionized the treatment of non-small-cell lung cancer (NSCLC). Even as an increasing number of immune checkpoint inhibitors and antibody–drug conjugates gained FDA approval for the remarkable response and durable remission they imparted to some patients, persistent challenges of primary and acquired resistance, imperfect predictive biomarkers, and toxicity remain. **Methods**: This review identifies and summarizes the most significant emerging clinical data being reported for immunotherapies in NSCLC. **Results**: We found that the most recent clinical trials are centered on antibody–drug conjugates, bispecific and multispecific antibodies, cellular therapies, and immunocytokines, while other ongoing trials continue to explore new monoclonal antibodies as well as a variety of immunotherapy modalities. **Conclusions**: Immunotherapy in NSCLC remains an area of rapid advancement, with the potential for further improvements in safety and efficacy as monotherapy and in multimodal approaches.

## Simple Summary

Immunotherapy has reshaped the treatment landscape of non-small-cell lung cancer, yet significant challenges such as treatment resistance, suboptimal predictive biomarkers, and toxicity persist. This review summarizes the most recent and emerging clinical data across a broad range of immunotherapeutic approaches, including antibody–drug conjugates, bispecific and multispecific antibodies, cellular therapies, and immunocytokines. By consolidating these rapidly evolving data, this review aims to provide context for ongoing research efforts and support the continued refinement of immunotherapy strategies in NSCLC.

## 1. Introduction

In 2026, more than 2,114,000 new cancer cases and over 626,000 cancer deaths are projected to occur in the United States [1]. Despite remarkable advances in early detection, targeted therapies, and immunotherapy over the past two decades, cancer remains the second leading cause of death in the United States and a leading cause of mortality worldwide. Globally, the cancer burden is even more staggering. According to the most recent estimates, there were close to 20 million new cancer diagnoses and 9.7 million cancer deaths worldwide in 2022 [2]. Lung cancer alone is expected to account for about 2.5 million new cases worldwide and 230,000–240,000 new cases annually in the United States [2,3,4]. About 85% of these cases in the United States are non-small-cell lung cancer (NSCLC), with the remainder being small-cell lung cancer (SCLC) [4]. The heterogeneity of cancer spanning histologically and molecularly distinct subtypes means that progress against one malignancy does not automatically translate to another, demanding broad and diversified cancer research. Among all malignancies, lung cancer exemplifies both the challenges and opportunities in cancer research. Even as lung cancer therapeutics face continued challenges from tumor heterogeneity, immune resistance, and a lack of sufficiently predictive biomarkers, lung cancer mortality has demonstrated a remarkable decrease over the last two decades, owing in part to rapid advances in treatment [1]. These advances have been driven by the discovery of treatable driver mutations (leading to targeted therapies) as well as the development of immune checkpoint inhibitors (ICIs) and other forms of immunotherapies.

**Figure 1:**
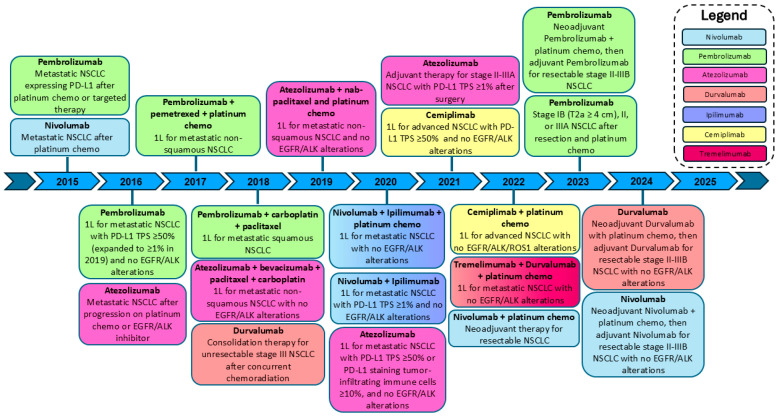
Timeline of approved immunotherapies for use in treatment of NSCLC. 1L: first line. TPS: tumor proportion score. Therapies approved in combination are represented visually with color gradients between their corresponding colors in the legend.

Immunotherapy drugs activate an individual’s own immune system to fight cancer. The first approved immunotherapy for NSCLC was nivolumab, an anti-PD-1 monoclonal antibody initially approved by the FDA in 2015 as a second-line treatment of metastatic squamous NSCLC [5]. In 2016, pembrolizumab (anti-PD-1 monoclonal antibody) became the first immunotherapy to gain FDA approval as a first-line therapy for NSCLC [6]. Since then, multiple other ICIs have been FDA-approved as first-line therapies, ICIs have gained approval for increasingly localized or early-stage disease, and the arsenal of FDA-approved ICIs now includes multiple other immune checkpoint inhibitors in addition to nivolumab and pembrolizumab: atezolizumab, durvalumab, cemiplimab, ipilimumab (in combination with nivolumab), and tremelimumab (in combination with durvalumab and chemotherapy). The timeline of FDA approvals of these drugs is shown in Figure 1 [5,6,7,8,9,10,11,12,13,14,15,16,17,18,19,20,21,22,23,24,25] (adapted from Leone et al. [26]).

For patients without biomarkers that would make them candidates for targeted therapies, these ICIs offer improved survival and response rates compared to conventional chemotherapy, with the benefit of relatively fewer severe adverse events. For example, in the KEYNOTE-024 trial, pembrolizumab monotherapy for advanced NSCLC with a PD-L1 tumor proportion score (TPS) ≥ 50% demonstrated a median overall survival (OS) of 30.0 months versus 14.2 months for platinum-based chemotherapy (HR 0.63), and a 5-year OS rate of 31.9% versus 16.3% for chemotherapy [27,28]. The objective response rate (ORR) was 44.8% for pembrolizumab versus 27.8% for chemotherapy. In total, 27% vs. 53% of patients had grade ≥ 3 treatment-related adverse events (TRAEs) [27]. For patients with lower TPS, ICIs remain the first-line treatment but are recommended to be used in combination with chemotherapy rather than as monotherapy, as clinical trial data have not shown a statistically significant OS benefit of ICI monotherapy over chemotherapy alone; however, combination immunotherapy + chemotherapy is still able to provide remarkable benefit in patients with NSCLC regardless of TPS, including <1% [29]. For example, a pooled analysis of randomized phase III trials testing pembrolizumab + chemotherapy in patients with advanced/metastatic nonsquamous (KEYNOTE-189) and squamous (KEYNOTE-407, KEYNOTE-021) NSCLC showed a median OS of 15.4 months with combination therapy vs. 10.6 months with chemotherapy, and 5-year OS rates of 12.5% vs. 9.3% [30].

Although ICIs lead to durable clinical benefit in some patients, others derive little or no sustained response. Treatment failure arises from primary or acquired resistance mechanisms [31]. Primary resistance refers to early disease progression occurring shortly after ICI initiation and reflects pre-existing tumor-intrinsic features or an immunosuppressive tumor microenvironment that prevents effective immune activation. In contrast, secondary resistance develops after an initial clinical response, as tumors adapt under immune selective pressure through genetic and epigenetic alterations that enable immune escape. These resistance mechanisms underscore a critical unmet need for next-generation therapeutic strategies to address three core goals: (1) overcoming immune resistance; (2) expanding the number of patients who benefit from immunotherapy beyond PD-1 responders; and (3) reducing dependence on PD-L1 as a biomarker.

Current biomarkers have variable correlation with clinical outcomes, even in drugs with known targeted mutations or pathways, and no single biomarker achieves adequate predictive accuracy for immunotherapy response in NSCLC. Although PD-L1 TPS and tumor mutational burden (TPS) remain among the most clinically validated and widely utilized biomarkers for immunotherapy selection, their predictive performance is generally moderate and insufficient as standalone tools. In addition, PD-L1 predicts the overall response rate, progression-free survival (PFS), and OS in non-squamous NSCLC, but not in squamous NSCLC. As another example, TROP-2 (trophoblast cell-surface antigen-2) is associated with poor overall survival and poor disease-free survival across solid tumors, including lung cancer, but does not reliably predict response to TROP-2-targeted antibody–drug conjugates designed to target it [32,33], although emerging data is showing that a computational biomarker, the normalized membrane ratio (NMR), may predict an improved response to the TROP-2-targeting Datopotamab deruxtecan [34].

While more research continues to be done on selecting and optimizing biomarkers for the application of existing therapeutics, continuing to target alternative immune pathways and resistance-associated tumor biology will be essential to enhancing the depth and durability of immunotherapeutic responses for more patients, especially nonresponders to classical immune checkpoint inhibitors. To those ends, the scientific community is actively exploring additional monoclonal antibodies, adoptive cell therapies (e.g., chimeric antigen receptor T-cell therapy (CAR-T), tumor-infiltrating lymphocytes (TIL)), and other cellular therapies, cancer vaccines, co-stimulatory antibodies, cytokine-based therapies, and microbiome-based therapeutics, among others. In addition, there is growing interest in bispecific antibodies and antibody–drug conjugates (ADCs) within the NSCLC therapeutic landscape. While these latter classes are sometimes considered targeted therapies rather than strictly immunotherapy, many monoclonal antibody-based therapies share the ability to engage the immune system’s ability to recognize tumor-associated antigens and are therefore included as part of this review.

This paper aims to review the most recently published data on emerging immunotherapies and antibody-derived therapies for advanced or metastatic NSCLC. A literature search was conducted utilizing American Society of Clinical Oncology (ASCO) 2025 meeting abstracts, American Association for Cancer Research (AACR) 2025 meeting abstracts, Society for Immunotherapy of Cancer (SITC) 2024 meeting abstracts, and ClinicalTrials.gov to identify phase I or seamless phase I/II clinical trials for advanced or metastatic NSCLC. All clinical trials with a mention of enrollment of patients with NSCLC and a start date of January 2024 to September 2025 were individually reviewed, and a literature search was conducted for each to identify corresponding preliminary data. Drugs included for discussion in the body were those that either had reported data from one of the studies identified or those for which data had been recently published at the aforementioned meetings in 2024/2025 and included at least one patient with NSCLC, regardless of study start date.

## 2. Antibody–Drug Conjugates

Antibody–drug conjugates are among the most rapidly expanding therapeutic classes in advanced NSCLC, with multiple early-phase agents showing promising activity in heavily pretreated patients. This class of targeted therapeutics combines the specificity of monoclonal antibodies with the potent cytotoxicity of small-molecule payloads, connected via engineered chemical linkers [35]. This “smart missile” approach enables selective delivery of highly cytotoxic agents directly to tumor cells expressing specific surface antigens, thereby broadening the therapeutic window compared to conventional chemotherapy while minimizing off-target toxicity to healthy tissues [36].

Of note, this strategy is also employed by the related emerging class of aptamer-conjugated drugs, which uses aptamers, short strands of DNA or RNA that fold into 3D structures that bind to molecular targets on cancer cells, instead of antibodies. Aptamer-conjugated drugs remain primarily in preclinical development, with the exception of AS1411 (a nucleolin-targeting aptamer), which has been tested in phase I/II trials but has limited data in NSCLC [35].

ADCs have a relatively robust body of clinical data. Across trials, the most common toxicities of ADCs include cytopenias and gastrointestinal adverse events. Interstitial lung disease (ILD) remains a clinically important risk, particularly with topoisomerase I inhibitor payloads that now dominate the ADC landscape.

Current antigenic targets of ADCs in NSCLC under development include HER2 (human epidermal growth factor receptor 2), HER3 (human epidermal growth factor receptor 3), TROP-2, DLL3 (Delta-like 3), CEACAM5 (carcinoembryonic antigen-related cell adhesion molecule 5), and MET (mesenchymal–epithelial transition factor) [37]. In NSCLC, three ADCs have already received FDA approval as of February 2026:

• Trastuzumab deruxtecan, an HER2-directed ADC approved for unresectable or metastatic NSCLC with activating HER2 mutations [38];
• Datopotamab deruxtecan, a TROP2-directed ADC approved for locally advanced or metastatic EGFR (epidermal growth factor receptor)-mutated NSCLC following prior EGFR-directed therapy and platinum-based chemotherapy [39];
• Telisotuzumab vedotin, a c-MET-directed ADC approved for non-squamous NSCLC with high c-MET protein overexpression (≥50% of tumor cells with 3+ immunohistochemical (IHC) staining) and EGFR wild-type status after prior systemic therapy [40].

More are used off-label or are in late-phase clinical trials. In this review, we focus on the emerging data for newer ADCs still in early-phase clinical trials. The bulk of these continue to target the aforementioned biomarkers that have shown promise as alternatives to PD-1/PD-L1, while two novel agents target PD-L1 with the potential to use an established biomarker to guide direct cytotoxic therapy in patients who may not experience immune activation with ICIs. Antibody-drug conjugates in early-stage clinical trials with published outcomes are listed below, while those without published outcomes are presented in Table 1.

### 2.1. 7MW3711

7MW3711 is a novel B7-H3-targeting ADC conjugated to a topoisomerase I inhibitor. It is designed to target B7-H3, an immune checkpoint molecule that is selectively expressed in a variety of solid tumors, including glioblastoma, ovarian cancer, NSCLC, and others. It primarily functions as a negative immunoregulatory protein and additionally activates signaling pathways such as ERK (Extracellular Signal-Regulated Kinase), PI3K (phosphatidylinositol 3-kinase), and STAT3 (Signal Transducer and Activator of Transcription 3) in cancer cells, promotes angiogenesis, and regulates cancer cell metabolism, ultimately leading to accelerated tumor growth [41]. Multiple other B7-H3-targeting ADCs are currently under early clinical investigation in solid tumors, including in NSCLC and SCLC [42,43,44]. In a first-in-human phase I/II study (NCT06008379), 37 patients with advanced lung cancer received at least one dose of 7MW3711 monotherapy, including 16 patients with SCLC and 21 patients with NSCLC, by the date cutoff on 8 January 2025 [45]. Five patients experienced dose-limiting toxicities, including decreased platelet count, decreased neutrophil count, myelosuppression, and decreased appetite. The maximum tolerated dose (MTD) had not yet been determined. Grade ≥ 3 treatment-related adverse effects (TRAEs) occurred in about 5% of patients. At the 4.5 mg/kg dose or above, nine partial responses (PRs) were observed, ORR was 36.0%, and disease control rate (DCR) was 96.0%. For squamous cell NSCLC (Sq-NSCLC) specifically, the ORR and DCR were 3/8 (37.5%) and 7/8 (87.5%), respectively. For SCLC, ORR and DCR were 62.5% and 100%. Bolstered by these findings of adequate tolerability and promising efficacy, the trial is currently continuing with the dose expansion phase [46].

### 2.2. DB-1311/BNT324

DB-1311/BNT324 is also an ADC directed at B7-H3 with a topoisomerase I inhibitor payload. It was tested in a global phase I/IIa clinical trial (NCT05914116) as monotherapy in heavily pretreated patients in various solid tumor types, including NSCLC, SCLC, castration-resistant prostate cancer (CRPC), and squamous cell carcinoma of the head and neck (SCCHN) [45]. The most common TRAEs were nausea, cytopenias, and decreased appetite, though the rates were not published. Patients with non-squamous NSCLC (*n* = 41) had an unconfirmed ORR (uORR) of 22%, while patients with squamous NSCLC (*n* = 25) had a uORR of 16% [45]. The drug also showed promise in SCLC in the same trial. Additional phase I/II and phase II trials are ongoing.

### 2.3. YL201

YL201 is another B7-H3-targeting ADC carrying a novel topoisomerase I inhibitor payload. It was studied in a global, multicenter phase I/Ib trial (NCT05434234 and NCT06057922) enrolling 312 heavily pretreated patients over multiple tumor types, of which 68 were patients with NSCLC without actionable genomic alterations [47]. In total, 97.1% of patients experienced TRAEs, with the most common being cytopenias, anorexia, nausea, and hypoalbuminemia. Grade ≥ 3 TRAEs occurred in 54.5% of patients, and treatment-related severe adverse events (SAEs) were reported in 29.2% of patients. Most of the 17 treatment discontinuations (5.4%) were due to hematological toxicities manageable with supportive care; however, eight deaths (2.6%) overall were considered related to YL201. The risk of pulmonary toxicity (specifically ILD) was low, at 1.3%. Among 64 evaluable wild-type NSCLC patients, ORR was 34.4%, driven heavily by the response in patients with lymphoepithelioma-like carcinoma (LELC). In these early data, YL201 also performed fairly well in the adenocarcinoma subset but poorly in the squamous NSCLC subset; ORR for patients with adenocarcinoma and squamous NSCLC were 28.6% and only 8.3%, respectively. DCR was 78.1% overall, with 78.6% DCR in adenocarcinoma and 58.3% DCR in squamous NSCLC [48]. In NSCLC, a phase I/II trial of YL201 in combination with Ivonescimab is underway.

### 2.4. DB-1310

DB-1310, a novel anti-HER3-directed ADC carrying a DNA topoisomerase I inhibitor payload, is another ADC currently in early clinical studies. HER3, or human epidermal growth factor receptor 3, is a signaling partner within the EGFR/HER family that dimerizes with other family members to activate the PI3K-AKT(Ak strain transforming)-mTOR (mechanistic target of rapamycin) pathway, promoting cell growth, survival, migration, and metabolic regulation [47]. A phase I/IIa dose escalation and expansion trial (NCT05785741) enrolled 123 patients as of 17 January 2025 [49]. In total, 65% of these were NSCLC patients. In total, 38/123 patients experienced ≥3 TRAEs, most commonly nausea, anemia, and decreased neutrophil or platelet counts. Interstitial lung disease was a notable side effect, occurring as grade 1 or 2 in seven patients (5.7%). The most promising antitumor activity was seen in the *EGFR*-mutated (EGFRm) NSCLC subgroup, where uORR was 35.7% compared to 25.5% across all tumor types. DCR was 80.9% across all tumors and 90.5% for EGFRm NSCLC. Median PFS was 5.4 months overall and 7.0 months for EGFRm NSCLC [49].

### 2.5. BNT326

BNT326, also called YL202, is an anti-HER3 ADC linked to eight molecules of YL0010014, a novel topoisomerase I inhibitor. A phase I trial (NCT05653752) tested BNT326 in pretreated patients with advanced/metastatic EGFR-mutated NSCLC or hormone receptor-positive (HR^+^) HER2^−^ breast cancer (BC) [50]. As of the date cutoff of 4 February 2024, 52 patients were enrolled (39 NSCLC, 13 BC). One DLT (grade 3 febrile neutropenia) occurred at the highest dose. TRAEs were common, with cytopenias, nausea, decreased appetite, vomiting, dry mouth, and fatigue occurring in 25–71% of patients, in descending order of frequency. The most notable grade ≥ 3 TRAEs were decreased WBC count (31% of patients), decreased neutrophil count (29%), decreased lymphocyte count (23%), and anemia (20%). In 46 patients evaluable for efficacy, ORR was 37% and DCR was 93.5% across all tumor types and dose levels, with no specific mention of data stratified by NSCLC tumor type. Because there were three treatment-related deaths at higher doses, the FDA placed a partial clinical hold in July 2024 that was subsequently lifted a few months later with the stipulation that the amended protocol would focus on doses no higher than 3 mg/kg, where the safety profile was judged “manageable” and encouraging activity was still observed. Efficacy data stratified by dose level was not publicly available. Multiple phase I/II and II trials are currently ongoing [50,51].

### 2.6. SHR-1826

SHR-1826 is a c-MET-directed ADC carrying a topoisomerase I inhibitor payload. c-MET is a receptor tyrosine kinase, encoded by the *MET* proto-oncogene, whose activation triggers downstream signaling pathways such as MAPK (Mitogen-Activated Protein Kinase), PI3K /mTOR, and STAT3 (signal transducer and activator of transcription 3), which are involved in the regulation of cell survival and proliferation, migration and invasion, angiogenesis, and epithelial-to-mesenchymal transition. High c-MET protein overexpression (IHC 3+ in ≥50% of tumor cells) is found in about 25% of NSCLC [52]. *MET* gene mutations, including exon 14 skipping and *MET* amplification, occur in 3–5% of NSCLC (mainly adenocarcinoma) and show limited overlap with c-MET protein overexpression [52,53]. A first-in-human, phase I trial (NCT06094556) studied SHR-1826 as monotherapy in 116 patients (72 of which were NSCLC patients) with *MET* alterations (overexpression, amplification, or activating mutation) as of 5 December 2024 [54]. One dose-limiting toxicity (DLT) was observed at the highest dose (grade 3 febrile neutropenia), and grade ≥ 3 TRAEs were reported in 56 (48.3%) patients, with the most common being decreased neutrophil count, decreased white blood cell (WBC) count, anemia, and decreased platelet count. Interstitial lung disease was a notable adverse effect, occurring in three (2.6%) patients, of which one was grade 3. Two (1.7%) patients discontinued treatment due to TRAE, and there were no treatment-related deaths. Among 58 evaluable patients with NSCLC, ORR was 39.7% and DCR was 94.8%. Response was not limited to patients with high c-MET expression levels or EGFR mutations. Median PFS among all 72 NSCLC patients was 6.8 months [54]. Multiple phase I/II and phase II trials began in late 2024 or 2025 and are currently recruiting patients with NSCLC and other solid cancers.

### 2.7. MYTX-011

MYTX-011 is a c-MET-targeting ADC carrying an MMAE (monomethyl auristatin E) payload. The first-in-human, multicenter phase I KisMET-01 trial (NCT05652868) studied MYTX-011 monotherapy in patients with previously treated, locally advanced or metastatic NSCLC [55]. TRAEs of any grade occurred in 90% of patients, and grade ≥ 3 TRAEs occurred in 48% of patients. The most common TRAEs were blurred vision (49%), keratopathy (44%), and nausea (29%). ORR was 38% in c-MET+ patients; DCR at 6 weeks/12 weeks/24 weeks was 97%/83%/53%, respectively. ORR was 44% in c-MET+ non-squamous EGFR-wild-type patients (*n* = 16), 38% in non-squamous EGFR-mutant patients (*n* = 8), and 25% in squamous cell carcinoma (*n* = 4) [55].

### 2.8. HLX43

HLX43 is a novel anti-PD-L1 ADC conjugated to a topoisomerase I inhibitor that has demonstrated promising results. A first-in-human phase I clinical trial (NCT06115642) enrolled patients with advanced/metastatic solid tumors, including NSCLC [56]. As of the data cutoff date of 28 June 2025, 56 patients with squamous NSCLC and 27 patients with non-squamous NSCLC were enrolled, most of whom were heavily pretreated. About half of the patients with non-squamous NSCLC had EGFR mutations. The most common grade ≥ 3 TRAEs were anemia (19.6%), decreased WBC count (19.6%), decreased neutrophil count (16.1%), and decreased lymphocyte count (12.5%). Immune-related AEs were reported in 21.4% of patients. Of the 54 response-evaluable patients, ORR was 37% and DCR was 87%. HLX43 demonstrated similar ORR (approximately 46%) in EGFR-mutated and EGFR wild-type non-squamous NSCLC, and also demonstrated notable efficacy in squamous NSCLC, with an ORR of 28.6% in heavily pretreated patients with squamous NSCLC (fourth line or later) compared to the standard therapy of docetaxel (ORR 12.8%). Further, HLX43 demonstrated similar efficacy between PD-L1-negative (TPS < 1%, *n* = 21) patients and PD-L1 TPS ≥ 1% patients [56,57].

### 2.9. PF-08046054 (PDL1V)

PF-08046054 (PDL1V) is an ADC that delivers MMAE to cells expressing PD-L1. A phase I clinical trial (NCT05208762) enrolled patients with relapsed or refractory solid tumors, including NSCLC, whose disease had progressed on standard-of-care therapies [58]. As of the date cutoff of 20 December 2024, 92 patients had been treated, including 30 patients with NSCLC. No DLTs were observed. The most common grade 1–2 TRAEs were peripheral neuropathy (27.2%), nausea (25.0%), diarrhea (23.9%), and fatigue (21.7%), and the most common grade ≥ 3 TRAE was anemia (5.4%). The incidence of immune-related adverse events (AEs) was 14.1%, all grade 3 or less. Confirmed ORR (cORR) across all patients with NSCLC was 26.7%, with a mean duration of confirmed response of 7.8 months [58]. This trial is still ongoing, with another phase III trial recently initiated to compare PDL1V against docetaxel monotherapy in pretreated, PD-L1-positive patients with NSCLC.

**Table 1:** Trials in progress without published outcomes for novel antibody–drug conjugates.

Drug	Target	NCT Number	Phase	Study Status	Combination or Monotherapy
JSKN016	TROP2 and HER3	NCT06868732	Ib	Not yet recruiting	Combination with multiple
TUB-040	NaPi2b	NCT06303505	I/II	Recruiting	Monotherapy
MGC028	ADAM9	NCT06723236	I	Recruiting	Monotherapy
PHN-012	Undisclosed	NCT07127874	I	Not yet recruiting	Monotherapy
M9140 (Precemtabart tocentecan)	CEACAM5	NCT06710132	Ib/II	Recruiting	Monotherapy
XB010	5T4	NCT06545331	I	Recruiting	Pembrolizumab
ALX2004	EGFR	NCT07085091	I	Recruiting	Monotherapy
PF-08046037	PD-L1 and TLR7a	NCT06974734	I	Recruiting	Sasanlimab
MBRC-201	Undisclosed	NCT07145255	I/II	Not yet recruiting	Monotherapy
BL-M09D1	Undisclosed	NCT07056556	I	Recruiting	Monotherapy
PF-08046876	integrin αvβ6 (ITGB6)	NCT07090499	I	Not yet recruiting	Monotherapy
LY4052031	Nectin-4	NCT06465069	I	Recruiting	Monotherapy
ENV-501	HER3	NCT06956690	I/II	Recruiting	Monotherapy
BA1302	CD228 (membrane-bound form)	NCT06596915	I	Recruiting	Monotherapy
LNCB74	B7-H4	NCT06774963	I	Recruiting	Monotherapy
ALE.P02	Claudin-1 (CLDN1)	NCT06747585	I/II	Recruiting	Monotherapy
PF-08046032	CD25	NCT06870487	I	Recruiting	Sasanlimab
BL-M17D1	HER2	NCT06714617	I	Recruiting	Monotherapy
LY4101174	Nectin-4	NCT06238479	I	Recruiting	Monotherapy
MGC026	B7-H3	NCT06242470	I	Recruiting	Monotherapy
SHR-1826	c-MET	NCT06844474	II	Recruiting	Monotherapy
LY4170156	FOLR1	NCT06400472	I	Recruiting	Bevacizumab, carboplatin

## 3. Bispecific Antibodies

Bispecific antibodies are an emerging class of engineered therapeutics designed to bind two distinct targets simultaneously, enabling coordinated biological effects that conventional monoclonal antibodies cannot achieve [59,60]. Their principal therapeutic advantage lies in enforced proximity: bispecific antibodies can physically bridge immune effector cells to tumor cells, simultaneously inhibit parallel oncogenic pathways, or localize immune activation within the tumor microenvironment, thereby generating synergistic antitumor activity that may surpass the effects of single-target antibodies or combination regimens administered separately [59,60,61]. These properties may help overcome mechanisms of resistance such as inadequate antigen presentation, pathway redundancy, and spatial separation of immune and tumor cells [60,61].

However, clinical implementation remains constrained by manufacturing and production complexity, variable tissue penetration in solid tumors, and safety concerns including cytokine release and on-target off-tumor toxicity resulting from potent immune activation [59,62,63]. Additional challenges in solid malignancies include antigen heterogeneity and immunosuppressive tumor microenvironments that limit durable responses [60,63]. Current research efforts are focused on improving tumor selectivity, optimizing pharmacokinetics, and combining bispecific antibodies with other platforms to enhance efficacy [60,62,63]. Important variations on the theme of bispecific antibodies include BiTEs (bispecific T-cell engagers), which bind any highly expressed structure on the surface of target cells with one arm and specifically CD3 on T-cells with the other, and multispecific antibodies targeting more than two targets simultaneously.

Since BiTEs first found success in hematologic cancers, especially in the treatment of various forms of lymphoma or multiple myeloma, multiple bispecific antibodies have gained FDA approval for solid tumors. Tarlatamab-dlle, a DLL3 × CD3 BiTE used against SCLC, was the first BiTE approved in a major solid tumor. In the treatment of NSCLC, amivantamab-vmjw is an EGFR × c-MET bispecific IgG that was first granted accelerated approval in May 2021 for NSCLC with *EGFR* exon 20 mutations in combination with or following platinum-based chemotherapy [64]. Zenocutuzumab-zbco is an HER2 × HER3 bispecific antibody approved in 2024 for advanced NRG1 (neuregulin-1)-fusion positive NSCLC or pancreatic adenocarcinoma [65]. Additional therapies in the pipeline, such as IBI318 (PD-1 × PD-L1 bispecific), demonstrate the ability to overcome immune resistance even in patients who were resistant to conventional anti-PD-1 therapy, and explore combinations of other immune checkpoint, angiogenesis, and oncogenic driver targets. Bispecific antibodies in early-stage clinical trials with published outcomes are listed below, while those without published outcomes are presented in Table 2.

### 3.1. ZGGS15

ZGGS15 is a humanized bispecific antibody of LAG-3 and TIGIT. LAG-3, or lymphocyte activation gene 3, also known as CD223, is an immune checkpoint receptor whose function is to regulate T-cell proliferation, activation, and effector function, contributing to immune suppression [66]. It received FDA approval for use in melanoma in 2022 as the combination drug Opdualag (relatlimab (anti-LAG-3 monoclonal antibody (mAb)) + nivolumab) and has also been studied alone and in combination, most commonly with PD-1 blockade, across multiple other tumor types including NSCLC [67,68]. TIGIT, or T-cell immunoreceptor with Ig and ITIM domains, is another inhibitory immune checkpoint receptor with advanced clinical trials for NSCLC, again often in combination with PD-1/PD-L1 inhibitors [69]. As of 8 January 2025, a total of 22 patients had been enrolled in the dose escalation phase of a phase I trial (NCT05864573) [70]. No DLTs were observed; TRAEs occurred in 20 (90.1%) patients but only one patient experienced a grade 3 TRAE of decreased lymphocyte count. In the eight patients with lung adenocarcinoma, five (62.5%) had achieved stable disease (SD), including two patients who maintained SD over 36 weeks. Among the broader group of 17 patients with any cancer who had at least one post-baseline tumor scan, 6 had achieved SD (DCR of 35.3%) [70].

### 3.2. AFM24

AFM24 is a bispecific innate cell engager designed to bind EGFR on tumor cells and CD16A (also known as FcγRIIIa) on innate immune cells, activating antibody-dependent cellular cytotoxicity via NK cells and antibody-dependent cellular phagocytosis via macrophages. A phase I/IIa study (NCT05109442) tested AFM24 in combination with atezolizumab in heavily pretreated patients with NSCLC, with a date cutoff of 15 January 2025 [71,72]. Among the *EGFR*-mutated subgroup, 28 patients had received treatment for a mean duration of 21.7 weeks. The most common TRAE was infusion-related reactions in 64% of patients, of which one patient experienced a grade 3 reaction. In total, nine patients had ≥3 TRAEs, the most common of which was neutropenia. Among 22 response-evaluable patients, ORR was 23% (one confirmed response (CR), three partial responses (PRs), one unconfirmed PR), DCR of 64%, and there was tumor shrinkage in 50% of patients. Median PFS was 5.5 months (95% CI 1.9–not evaluable with a median follow-up of 9 months). Among the *EGFR*-wild-type subgroup, 43 patients had received treatment. Again, the most common TRAE was infusion-related reactions, in 54% of patients, of which four patients had a grade 3 reaction. The most common grade ≥ 3 TRAE was AST/ALT elevations, seen in two patients, both of whom had full resolution. Of the 35 response-evaluable patients, ORR was 23% (one complete response, seven partial responses), tumor shrinkage was achieved in 46% (16/35), and DCR was 77%. Median PFS was again 5.5 months (95% CI 2.9–7.4). Unfortunately, this study was terminated for financial reasons and no follow-up study is currently planned [71,72].

### 3.3. IBI363

IBI363 is a first-in-class PD-1/IL-2 bispecific antibody that blocks PD-1 and activates IL-2 signaling. A first-in-human phase I trial (NCT05460767) was conducted to study IBI363 as monotherapy in patients with advanced NSCLC who had failed or were intolerant of standard therapy [73]. As of the date cutoff of 6 December 2024, 136 NSCLC patients were enrolled. TEAEs occurred in 135 patients, of which grade ≥ 3 TEAEs were 42.6%. Nine of these led to treatment discontinuation and four led to death, with one of the latter being considered treatment-related (unexplained). The most common TEAEs were arthralgia (51.5%; 3.7% grade ≥ 3), anemia (43.4%; 3.7% grade ≥ 3), and rash (38.2%; 4.4% grade ≥ 3). The most encouraging results were reported in patients receiving 3 mg/kg every 3 weeks (Q3W). Among the 30 patients with squamous cell carcinoma treated at 3 mg/kg Q3W, ORR was 43.3%, confirmed ORR was 36.7%, DCR was 90.0%, and median PFS was 7.3% at a median follow-up of 7.3 months. In the 30 patients with adenocarcinoma with no actionable genomic alterations treated at 3 mg/kg Q3W, ORR was 28.0%, confirmed ORR was 24.0%, DCR was 76.0%, and median PFS was 4.2 months at a median follow-up of 5.3 months. In patients at all dose levels with a TPS < 1%, the ORR was 45.5% for squamous NSCLC (*n* = 22) and 29.4% for adenocarcinoma (*n* = 17). These data suggest good efficacy of IBI363 in NSCLC, especially in patients with the squamous subtype, with comparable efficacy for patients with low PD-1 expression [73]. Multiple phase II studies are underway to study IBI363 in NSCLC and in other advanced solid tumors, with no results from these published yet.

### 3.4. SMET12

SMET12 is a bispecific antibody and T-cell engager that binds EGFR on tumor cells and CD3 on T-cells to bring them into close proximity. A phase I study (NCT06208033) was conducted to study SMET12 in combination with toripalimab and chemotherapy in patients with *EGFR*-mutated advanced NSCLC [74]. As of the date cutoff of 21 January 2025, 27 of 31 patients were evaluable for efficacy. The most common types of grade ≥ 3 TRAEs were in line with those seen in other studies involving toripalimab and the various chemotherapy backbones tested, with leukopenia, pneumonia, immune-related adverse events, and anemia predominating, though frequencies were somewhat increased [75,76]. Efficacy was greatest in the treatment-naïve cohort, where ORR was 83.3% and DCR was 100%, with a median PFS of 8.3 months. This was followed by the cohort of patients who had failed prior TKI treatment, with ORR of 41.7%, DCR of 100%, and a median PFS of 7.2 months. The last cohort, consisting of patients resistant to first-line immune checkpoint inhibitor therapy, demonstrated the lowest but still meaningful response, with ORR of 22.2% and DCR of 66.7%, with a median PFS of 4.2 months [74].

**Table 2:** Trials in progress without published outcomes for multispecific antibodies. TAA: tumor-associated antigen. BiTE: bispecific T-cell engager.

Drug	Comments	Targets	NCT Number	Phase	Study Status	Combination or Monotherapy
RC148	Bispecific	PD-1 and VEGF	NCT06883630	Ib	Recruiting	Chemotherapy
BC008-1A	Bispecific	PD-1 and TIGIT	NCT06773507	I	Recruiting	Monotherapy
BGB-B2033	Bispecific	GPC3 and 4-1BB	NCT06427941	I	Recruiting	Tislelizumab
YH32364	Bispecific	EGFR and 4-1BB	NCT06975410	I/II	Recruiting	Monotherapy
AK129	Bispecific	PD-1 and LAG-3	NCT06943820	Ib/II	Recruiting	Chemotherapy
PF-07826390	Bispecific	LILRB1 (also known as ILT2) and LILRB2 (ILT4)	NCT06546553	I	Active, not recruiting	Sasanlimab, standard of care
DR-0202	Bispecific	CLEC7A (Dectin-1) and undisclosed TAA	NCT06999187	I	Recruiting	Monotherapy
CTX-8371	Bispecific	PD-1 and PD-L1	NCT06150664	I	Recruiting	Monotherapy
DS-2243a	Bispecific; BiTE	HLA-A*02/NY-ESO-1 complex and CD3	NCT06644755	I	Recruiting	Monotherapy
GNC-077	Tetraspecific	CD3 and undisclosed TAAs	NCT06612840	I	Recruiting	Monotherapy
MDX2001	Tetraspecific	CD3, CD28, TROP2, and c-MET	NCT06239194	I/II	Recruiting	Monotherapy
KJ015	Bispecific	HER2	NCT07036185	I	Not yet recruiting	Monotherapy

## 4. Monoclonal Antibodies

Even as the PD-1/PD-L1 space has become saturated, new immune checkpoint inhibitors are under development as alternatives to this pathway and to reduce dependence on PD-L1 as a biomarker. Additionally, while the class of original monoclonal antibodies has given rise to more sophisticated platforms and combination strategies, including bispecific/multispecific antibodies and ADCs, monoclonal antibodies continue to undergo innovation in structure (e.g., nanobody formats, Fc engineering) and novel targets, including biomarker-agnostic epitopes. Monoclonal antibodies in early-stage clinical trials with published outcomes are listed below, while those without published outcomes are presented in Table 3.

### GV20-0251

GV20-0251 is a monoclonal antibody against IGSF8 (immunoglobulin superfamily member 8), a novel immune checkpoint that is broadly expressed across solid tumors, including NSCLC, colorectal cancers, breast cancer, and melanoma. Its antitumor effect is proposed to be mediated by the release of NK cell cytotoxicity and enhancement of dendritic cell antigen presentation to improve T-cell infiltration and activity [77]. A phase I/IIa trial (NCT05669430) was conducted to study GV20-0251 as a monotherapy in patients with advanced solid tumors [78]. Currently available data were obtained from a cohort of primarily melanoma patients (17), in addition to four NSCLC and one cervical cancer patients. Of 42 enrolled patients, none had DLT, and TRAEs occurred in 55% of patients, predominantly grade 1 or 2, with a single grade 3 event of pneumonitis. One of the four enrolled NSCLC patients demonstrated tumor shrinkage, though this did not appear to meet criteria for a partial response, as the authors reported no responses were seen in patients with tumor types other than melanoma [78]. As such, GV20-0251 is less likely to be promising as an NSCLC treatment; however, other phase I and I/II trials are still underway and more data is needed.

**Table 3:** Trials in progress without published outcomes for monoclonal antibodies.

Drug	Target	NCT Number	Phase	Study Status	Combination or Monotherapy
WM-A1-3389	IGSF1	NCT05872867	I	Recruiting	Pembrolizumab
BT-02	ITPRIPL1	NCT07110363	I	Not yet recruiting	Monotherapy
ZM008	LLT1	NCT06451497	I	Recruiting	Pembrolizumab
LM-108	CCR8	NCT06479759	II	Recruiting	Sintilimab
S095018, S095024, S095029	TIM3, CD73, NKG2A	NCT06162572	Ib/II	Recruiting	Cemiplimab
GV20-0251	IGSF8	NCT07070518	I/IIa	Recruiting	Monotherapy
LB-LR1109	LILRB1	NCT06332755	I	Recruiting	Monotherapy
TQB2928	CD47	NCT06585059	Ib	Not yet recruiting	Almonertinib
OSE-279	PD-1	NCT05751798	I/II	Recruiting	OSE2101 (cancer vaccine)

## 5. Microbiome-Based Therapeutics

Microbiome-based therapeutics represent an emerging class of interventions that modulate the gut microbial ecosystem to enhance cancer immunotherapy efficacy and overcome treatment resistance. These approaches include fecal microbiota transplantation (FMT), probiotics, prebiotics, dietary modifications, and engineered bacterial consortia designed to reshape the gut microbiome composition and function [79]. In a phase 2 trial in the first-line setting, FMT combined with immunotherapy has achieved objective response rates of 65–80% in melanoma and NSCLC [80]. Mechanistically, beneficial microbes enhance antitumor immunity through multiple pathways: promoting dendritic cell maturation and migration to tumors, increasing CD8^+^ T-cell activation and infiltration, producing immunomodulatory metabolites like short-chain fatty acids and indole-3-propionic acid, and modulating PD-1/PD-L1 expression [81,82].

Clinical translation has remained limited in part due to interpatient variability in microbial ecology, incomplete mechanistic understanding, challenges in standardization and manufacturing, and safety considerations related to microbial transfer and long-term ecological effects. In addition, most evidence so far has come from single-arm phase I/II studies. Future directions include developing precision microbiome interventions and creating defined bacterial consortia or engineered probiotics, in addition to conducting definitive phase 3 trials [83,84].

### 5.1. Leuconostoc mesenteroides

*Leuconostoc mesenteroides* is a lactic acid bacterium primarily used in the fermentation of foods such as kimchi, sauerkraut, and dairy products [85]. It has been studied for potential therapeutic applications as a probiotic for gastrointestinal and metabolic health, wound healing, and modulation of the immune response, although the bulk of this data is in vitro or in mouse models rather than in humans [85,86]. In a colon cancer cell line model, conditioned media or live bacteria induced apoptosis, inhibited proliferation, and upregulated PD-L1 with a paradoxical effect of activating T-cells, enhancing immunotherapy efficacy, and activating innate immune pathways via TLR2 (Toll-like receptor 2) and NOD2 (nucleotide-binding oligomerization domain-containing protein 2), among others [87,88]. A phase I/II trial studied CJRB-101 in combination with pembrolizumab in selected types of advanced or metastatic cancer, including NSCLC [89]. Among 32 total patients, no dose-limiting toxicity was observed; TRAEs occurred in 21.9%; and only 1 patient (3.2%) experienced grade > 3 TRAE, which was anemia. Of the 20 patients deemed efficacy evaluable, ORR was 44% for ICI-naïve, metastatic NSCLC (4/9), and DCR was 30% (3/10) for ICI-refractory NSCLC. Bulk RNA sequencing showed that patients who derived clinical benefit showed enrichment in T-cell activation and upregulation of innate and adaptive immune response compared to those with no clinical benefit [89].

### 5.2. Trials in Progress

One biotherapeutic product/bacterium was identified as actively undergoing study in a phase I/II trial in patients with advanced NSCLC refractory to immunotherapy. EXL01 contains an active agent of a single, unmodified strain of the gut commensal bacterium *Faecalibacterium prausnitzii*. *F. prausnitzii* triggers activation of the NOD2 signaling pathway, an innate immune-sensing pathway [90]. This pilot, single-arm trial is currently recruiting and testing EXL01 in combination with nivolumab.

## 6. Cellular Therapies

Cellular therapies represent a diverse class of adoptive immunotherapies that harness the patient’s own or donor-derived immune cells—including TILs, CAR-T, T-cell receptor-engineered T-cells (TCR-T), and natural killer (NK) cells—to directly target and eliminate tumor cells.

TIL therapy involves isolating lymphocytes already present within a patient’s tumor, expanding them ex vivo, and reinfusing them after lymphodepleting chemotherapy. Their inherently polyclonal makeup overcomes limitations of therapies targeting one or few molecules, including tumor heterogeneity and antigen escape. In one phase I trial of 20 patients with advanced NSCLC treated with nivolumab and TILs following initial progression on nivolumab monotherapy, TIL therapy achieved a partial response in 5 of 13 (38.5%) evaluable patients, and twos complete responses (15.4%) [91]. However, this class faces significant limitations of high manufacturing costs, procedural complexity, and substantial toxicity from lymphodepletion and IL-2 administration. In addition, despite the advent of this technology decades ago, data for TIL therapy in NSCLC remains limited; interest in this field had stalled due to manufacturing challenges and the concurrent rise in targeted therapy and checkpoint inhibitors, with only recently renewed attention.

CAR T-cells are genetically engineered to express chimeric antigen receptors that recognize specific tumor surface antigens (e.g., EGFR, MSLN (mesothelin), CEA (carcinoembryonic antigen), MUC1 (mucin-1), ROR1 (Receptor Tyrosine Kinase-Like Orphan Receptor 1), HER2 in NSCLC) independent of MHC presentation [92]. Having revolutionized the treatment of hematological malignancies, they unfortunately face challenges in solid tumors including a lack of tumor-specific antigens, antigen heterogeneity and escape, poor trafficking and persistence in tumor masses, and T-cell exhaustion within the immunosuppressive tumor microenvironment. This tumor microenvironment provides structural barriers including abnormal vasculature and changes in the extracellular matrix [93]; metabolic barriers including hypoxia and nutrient depletion [94,95]; and immunological barriers of increased immunosuppressive populations (e.g., regulatory T-cells, myeloid-derived suppressor cells (MDSCs), and tumor-associated macrophages (TAMs), as well as upregulated suppressive immune checkpoints and cytokines such as PD-L1, TGF-β, and IL-10) [96]. In NSCLC and other cancer types, various engineering innovations have been developed to try to optimize this technology [97,98]. At this time, existing outcomes of the limited CAR T-cell research in NSCLC have shown only modest clinical responses, though emerging data with CEA-targeted CAR T-cells appears more promising (see below).

TCR-T-cell therapy genetically engineers a patient’s T-cells to express a specific T-cell receptor that recognizes tumor-associated antigens presented on human leukocyte antigen (HLA) molecules. Unlike CAR T-cells, TCR-T-cells are not limited to surface antigens and can recognize peptides derived from the entire cellular proteome. In NSCLC, early clinical data have demonstrated that neoantigen-specific TCR-T-cells can produce partial responses in chemotherapy and immunotherapy-resistant patients, most notably using NY-ESO-1 TCR-T. However, TCR-T therapy faces similar challenges as CAR T-cell therapy, including limited antigen expression, tumor heterogeneity, and the immunosuppressive tumor microenvironment, in addition to HLA restriction, which significantly limits broad applicability.

Lastly, NK cell therapies leverage the innate cytotoxic capacity of NK cells to recognize and eliminate malignant cells independent of antigen presentation or T-cell receptor specificity. In contrast to TILs, CAR T-cell therapies, and TCR-T therapy, NK cells detect loss of major histocompatibility complex (MHC) expression, which is a common mode of tumor immune evasion. Critically, NK cells do not require HLA matching and exhibit minimal graft-versus-host disease, enabling “off-the-shelf” products that can be manufactured at scale and stored, reducing costs and treatment delays compared to autologous T-cell therapies [99]. Early-phase clinical studies in solid tumors, including NSCLC, have demonstrated favorable safety profiles with low rates of severe cytokine release syndrome compared with T-cell-based cellular therapies, although objective responses have been variable and generally modest to date. A 2025 meta-analysis of NK cell therapy in advanced NSCLC found that NK cell therapy demonstrated comparable disease control and 1-year survival to existing treatments [100]. Clinical efficacy is limited by poor tumor infiltration, limited persistence in vivo, and functional exhaustion in the tumor microenvironment. Cellular therapies in early-stage clinical trials with published outcomes are listed below, while those without published outcomes are presented in Table 4.

### 6.1. CAR T-Cells Targeting CEA

CEA, or carcinoembryonic antigen, also known as CEACAM5, is a cell-surface glycoprotein overexpressed in a wide range of epithelial cancers. Though most classically associated with colorectal and other gastrointestinal adenocarcinomas, it is also commonly and strongly expressed in medullary thyroid carcinoma, NSCLC, SCLC, and breast and ovarian cancers, among others [101]. CEA is strongly elevated in up to 74% of adenocarcinomas and is present in other histologic subtypes, although at a lower frequency and intensity [101]. Though CEA levels are not sufficiently specific to be used for routine screening or surveillance in NSCLC, elevated serum CEA levels before, during, or after treatment are poor prognostic signs, and its selective tumor expression and cell-surface localization make it an attractive target [102,103]. CAR T-cells targeting CEA were tested in solid tumors in a phase I trial (NCT06006390) in China, with an analysis on the subset of patients with NSCLC [104]. Fifteen patients received CAR T-cell infusion, with six patients receiving the maximum dose (3 × 10^6^ cells/kg). No dose-limiting toxicities, grade 4 cytokine release syndrome (CRS), or immune effector cell-associated neurotoxicity syndrome (ICANS) were observed. Little other information was provided about safety except that no adverse events were observed during a three-month safety evaluation. With a median follow-up of 5.7 months, seven patients achieved PR, six had SD, and the remaining two experienced progressive disease (PD). The DCR was 87% and ORR was 47%. Patients with ≥30% intense and complete CEA staining in tumor cells and no brain metastases had better PFS (72.7% vs. 25.0%) and OS than the comparison group (90.9% vs. 0%) [104].

### 6.2. sIL15_TRACK NK Cells/COH06

sIL15_TRACK NK cells, also called COH06, are unmatched, allogeneic, off-the-shelf PD-L1^+^ NK cells engineered to express soluble IL-15 (sIL15). Designing the NK cells to express sIL15 helps sustain the NK cells in vitro and in vivo before and after administration, respectively. sIL15_TRACK NK cells were studied as monotherapy in a first-in-human phase I trial (NCT05334329) of six patients (over two dose levels) with advanced NSCLC who had progressed on chemotherapy and ICI therapy [105]. Four of the six patients had grade 3 or 4 cytopenia associated with the preceding lymphodepletion regimen, but the sIL15_TRACK NK cells were well tolerated, with one grade 3 toxicity of sinus tachycardia; otherwise, there were grade 1–2 toxicities, and no infusion-related reactions, evidence of cytokine release syndrome, or CNS toxicity. In total, 3/6 (50%) of patients had stable disease at week 6 after initiation of therapy, and the other 3/6 (50%) experienced disease progression by week 6. This trial is ongoing, and although the NK cells were tested as monotherapy for the first part of the trial which has been reported on thus far, the investigators plan to add atezolizumab for additional patients. It is hypothesized that the mechanism by which patients with tumors lacking PD-L1 expression can respond to anti-PD-L1 mAb therapy may involve PD-L1^+^ NK cells in the tumor microenvironment, which degranulate and produce IFN-γ after being bound by anti-PD-L1 mAb [106,107].

### 6.3. A2B694

A2B694 is a CAR T-cell therapy targeting MSLN, a cell-surface glycoprotein whose expression is restricted to mesothelial cells in normal tissue but upregulated in a variety of solid tumors, including mesothelioma, ovarian, uterine, gastric, pancreatic, and lung cancers. Overexpression in these tumors appears to drive tumor proliferation, metastasis, resistance to therapy, and immune evasion [108]. A2B694 was designed with an inhibitor receptor for HLA-A*02, thereby increasing the specificity for tumor cells which have lost HLA expression and avoiding on-target off-tumor effects [109]. A first-in-human phase I/II trial (NCT06051695) treated five heavily pretreated patients (three ovarian cancer, one pancreatic cancer, and one lung adenocarcinoma) with lymphodepleting chemotherapy and then A2B694 [110,111]. The most common adverse events were decreased lymphocyte count (14.6%) and decreased appetite (12.5%). There were no DLTs, CRS, or ICANS. All five patients were efficacy evaluable, showing successful CAR T expansion, persistence, and tumor infiltration. The patient with NSCLC experienced a partial response at day 90 post-infusion [110,111]. The study is currently still in the process of recruiting additional patients.

**Table 4:** Trials in progress without published outcomes for cellular therapies. CAR-T: chimeric antigen receptor T-cell therapy. TCR-T: T-cell receptor-engineered T-cell therapy. TILs: tumor-infiltrating lymphocytes.

Drug	Type	Target	NCT Number	Phase	Study Status	Combination or Monotherapy
Anti-Trop2 Universal CAR-NK (U-CAR-NK)	CAR-T	TROP-2	NCT06454890	I/II	Not yet recruiting	Monotherapy
MOv19-BBz	CAR-T	Folate receptor-α (FRα/FOLR1)	NCT07116057	I	Not yet recruiting	Monotherapy
REVO-UWD-03	CAR-T	GPC3	NCT06653023	Early phase I	Recruiting	Monotherapy
A2B395	CAR-T	EGFR	NCT06682793	I/II	Recruiting	Monotherapy
EphA2-targeted CAR-DC and CAR-T-Cells	CAR-DC, CAR-T	EphA2	NCT06972576	I	Recruiting	Monotherapy
CRTKVA11	TCR-T	KRAS	NCT06767046	I	Not yet recruiting	Monotherapy
DCTY1102	TCR-T	KRAS	NCT07014878	I	Not yet recruiting	Monotherapy
AFNT-211	TCR-T	KRAS G12V	NCT06105021	I/II	Active, not recruiting	Monotherapy
NT-112 (AZD0240)	TCR-T	KRAS	NCT06218914	I	Recruiting	Monotherapy
Autologous T-cells modified to express TCRs recognizing KRAS + KRAS peptide vaccine	TCR-T, cancer vaccine	KRAS G12D or G12V mutations	NCT06253520	Ib	Recruiting	Monotherapy
HS-IT101	TILs		NCT07105176	Ib	Not yet recruiting	Monotherapy
GC101 TIL	TILs		NCT06473961	Ib	Recruiting	PD-1 antibody (unspecified)
Autologous TILs	TILs		NCT06538012	I/II	Recruiting	Pembrolizumab
C-TIL051	TILs		NCT05676749	I	Recruiting	NKTR-255, anti-PD1
KSQ-004EX	CRISPR-edited TILs	SOCS1/Regnase-1	NCT06598371	I/II	Recruiting	Cyclophosphamide, fludarabine, IL-2

## 7. Cancer Vaccines

Peptide/protein vaccines and mRNA vaccines deliver tumor-associated or tumor-specific antigen fragments or mRNA, respectively, to dendritic cells, which process and present these peptides on MHC molecules to activate tumor-specific CD8^+^ and CD4^+^ cells. These offer a favorable safety profile, high specificity, and ease of manufacturing, but are limited by low immunogenicity, poor stability, and the immunosuppressive tumor microenvironment. Combination with checkpoint inhibitors is required to prevent suppression of vaccine-induced T-cells [112]. Cancer vaccines in early-stage clinical trials without published outcomes are presented in Table 5.

### BNT116

BNT116 is a mRNA vaccine composed of six mRNAs, each encoding a tumor-associated antigen frequently expressed in NSCLC: CLDN6 (claudin-6), KK-LC-1 (Kita-Kyushu lung cancer antigen-1), MAGE-A3 (Melanoma antigen gene A3), MAGE-A4 (Melanoma antigen gene A4), MAGE-C1 (Melanoma antigen gene C1), and PRIME [113]. It was studied in the open-label, first-in-human LuCa-MERIT-1 phase I trial (NCT05142189) in combination with established therapies in patients with advanced or metastatic NSCLC. Preliminary results from this trial have recently been published, studying different populations of patients treated with BNT116.

The first published results were in April 2024, testing the combination of BNT116 and docetaxel in 20 patients with unresectable or metastatic NSCLC who had previously progressed on a PD-1 or PD-L1 inhibitor and platinum-based chemotherapy. In total, 7/20 patients (35%) had partial response, and 10/20 (50%) had stable disease, for an ORR of 35% and DCR of 85% [114].

In parallel, the combination of BNT116 + cemiplimab was studied in patients with advanced, PD-L1 ≥ 50% NSCLC progressing on or after anti-PD-1 or anti-PD-L1 therapy; results were published in November 2024. In total, 2/20 patients (10%) had a partial response and 14/20 patients (70%) had stable disease, resulting in an ORR of 10% and DCR of 80% [115].

Another analysis published in April 2025 studied BNT116 + cemiplimab in patients who were not eligible for standard first-line treatment with platinum-based chemotherapy due to frailty. Again, 20 patients had been treated, with 9/20 patients (45%) experiencing partial response, and 7/20 (35%) experiencing stable disease, for an ORR of 45% and DCR of 80% [113].

The safety data from these trials were consistent, with all patients experiencing at least one treatment-emergent adverse event (TEAE) (or as reported in the first and last analyses above, TRAE) attributable to BNT116. Approximately 15% (3/20) of patients experienced grade ≥ 3 TRAEs or serious TEAEs attributable to BNT116, with additional toxicity from the respective combination agents used in each trial. No DLTs or fatal TRAEs were reported [113,114,115]. A phase II trial (EMPOWERVAX Lung-1, NCT05557591) is underway to study BNT116 + cemiplimab vs. cemiplimab alone as a first-line treatment for advanced NSCLC.

**Table 5:** Trials in progress without published outcomes in patients with NSCLC for cancer vaccines.

Drug	Type	Target	NCT Number	Phase	Study Status	Combination or Monotherapy
PANDA-VAC	Peptide vaccine	Neoantigen peptide	NCT04266730	I	Not yet recruiting	Pembrolizumab
CVHNLC	mRNA vaccine	CV09070101	NCT07073183	I	Not yet recruiting	Pembrolizumab
ALK peptide vaccine	Peptide vaccine	ALK	NCT05950139	I/II	Recruiting	Monotherapy
Labvax 3(22)-23	Peptide vaccine	Labyrinthin	NCT05101356	I/II	Recruiting	GM-CSF +/− pembrolizumab
BMD006	mRNA vaccine	Six tumor-associated antigens	NCT06928922	I	Recruiting	Anti-PD-1 therapy or Ivonescimab
PNeoVCA	Peptide vaccine	Patient-specific neoantigens	NCT05269381	I/II	Recruiting	Cyclophosphamide and/or pembrolizumab
OVM-200	Peptide vaccine	Survivin	NCT05104515	I	Recruiting	Monotherapy
PNV21-001	Peptide vaccine	Patient-specific neoantigens	NCT05098210	I	Recruiting	Poly-ICLC and nivolumab

## 8. Other Molecules, Including Cytokines, Gene Therapy, and Radiolabeled Antibodies

### 8.1. SAR445877

SAR445877 is an antibody–cytokine fusion protein consisting of an Fc-silenced human anti-PD-1 monoclonal antibody fused to a mutated IL-15/IL-15Rα complex. The PD-1-targeting moiety inhibits downstream signaling of PD-1-expressing exhausted T-cells, and the IL-15 moiety activates IL-15-mediated signaling, stimulating proliferation of NK cells, cytotoxic T lymphocytes, and memory T-cells [116,117]. Preclinical studies demonstrated upregulation of cytotoxic pathways in vitro and superior efficacy to the respective single target agents in mouse models. A phase I/II study of SAR445877 in patients with advanced or metastatic solid tumors enrolled 32 patients [118]. In a report of the initial results, TRAEs were reported in 47% of patients, most commonly cytokine release syndrome, with 17 patients experiencing grade ≥ 3 TRAEs. Six patients discontinued treatment due to any TEAEs. Confirmed partial response was reported in seven patients, and stable disease ≥ 6 months was observed in six patients. Notably, an unspecified number of NSCLC patients were enrolled in the trial but none of these patients were reported to have a confirmed partial response. The phase I/II trial is still ongoing (NCT05584670) [118].

### 8.2. KB707

KB707 is a novel gene therapy that uses a replication-defective herpes simplex virus 1 (HSV-1)-based vector to deliver IL-12 and IL-2 to the local tumor microenvironment. A phase I/II study (NCT06228326) is investigating inhaled KB707 in advanced solid tumors involving the lungs, primarily NSCLC [119]. As of 8 January 2025, 39 patients were enrolled, with the maximum tolerated dose not reached and no grade 4 or 5 TRAEs observed. Among the 11 response-evaluable NSCLC patients, the ORR was 27% (3/11) and DCR was 73% (8/11). The median duration of response had not been reached, and treatment duration ranged from 10.3 to 33.3 weeks. These results were reported in patients who received KB707 as monotherapy. The study has since expanded to evaluate KB707 in combination with pembrolizumab, with or without chemotherapy, in advanced NSCLC patients [119].

### 8.3. DK2^10^

DK2^10^ is a tumor-targeted dual-cytokine immunocytokine that combines IL-2 and IL-10 into a single-chain fusion protein that is targeted to tumors via an EGFR-targeted single-chain variable fragment (scFv). It is being studied in a phase I trial (NCT05704985) against advanced tumors expressing EGFR, and 6 of the 35 patients enrolled at the time of data publication had NSCLC [120]. The most common TRAE was injection site reactions (63% of patients), but grade 3 TRAEs were most notable for fatigue (*n* = 4), anemia (*n* = 3), and syncope (*n* = 3). No DLTs were observed. In total, 33% of evaluable patients had a best ORR of SD [120].

### 8.4. [225Ac]Ac-AKY-1189

[225Ac]Ac-AKY-1189 is a radiolabeled antibody engineered to deliver actinium-225 to nectin-4-expressing tumor cells. Nectin-4 is overexpressed in urothelial, breast, head/neck, lung, colorectal, and cervical tumors, with restricted normal tissue expression that is primarily limited to embryonic tissues and skin [121]. In addition to its established roles in mediating cell adhesion and promoting tumor growth through PI3K/AKT signaling, nectin-4 has recently been suggested to function as a negative immune regulatory checkpoint [122]. Enfortumab vedotin (Padcev) is a nectin-4-targeted ADC that has been approved for urothelial carcinoma, but no therapy has yet been approved for NSCLC [123]. Multiple radiolabeled antibodies are currently being studied that target nectin-4; 68Ga was studied for human imaging feasibility, 177Lu was studied for dosimetry, and 225Ac is now being studied for therapeutic antitumor efficacy [124,125]. Of eight patients with various tumor types (including one with lung cancer) dosed with [177Lu]Lu-AKY-1189, none experienced adverse events, and there was no significant accumulation of [177Lu]Lu-AKY-1189 in healthy tissues. At the same time, there was robust uptake across multiple tumor types [125]. Based on these data, a phase I clinical trial (NCT07020117) to study the therapeutic effects of [225Ac]Ac-AKY-1189 in solid tumors including NSCLC is now recruiting.

### 8.5. Trials in Progress

Table 6 lists other therapeutics not falling into the categories above that are in early-stage clinical trials without published outcomes.

## 9. Conclusions and Future Directions

The field of immunotherapy has been a significant area of research in NSCLC and other malignancies. Thus far, immune checkpoint inhibitors have brought renewed hope to patients with previously difficult-to-treat malignancies, including advanced or refractory disease or those lacking targetable mutations. However, the response currently remains highly variable and limited to a minority of patients. More research is needed on overcoming/preventing ICI resistance, identifying predictive biomarkers, and reducing dependence on PD-L1, which has dominated the landscape thus far, minimizing and managing immune-related adverse events, developing additional immunotherapy approaches, and adopting personalized approaches to cancer treatment.

In recent years, ADCs, bispecific and multispecific antibodies, cellular therapies, and immunocytokines have been especially popular and promising areas of research. Additionally, even as established ICIs continue to demonstrate their utility in increasingly early-stage disease and neoadjuvant settings, new monoclonal antibodies are continuing to be studied, which still focus heavily on targeting novel immune checkpoints. Beyond this, immunotherapy research in NSCLC boasts a breadth encompassing many other classes of therapies, including immunomodulators, oncolytic viruses, antibody–drug conjugates, radiolabeled antibodies, anchored cytokine immunotherapy, antisense mRNA, gene therapy, cancer vaccines, and microbiome-based therapeutics, which offer hope for overcoming resistance to current treatments, expanding the proportion of patients who derive durable benefit beyond classic anti-PD-1/PD-L1 or anti-CTLA-4 responders, and enabling more precise, personalized, and combination-based therapeutic strategies in the future.

These early-phase clinical trial data collectively highlight the emerging drugs shaping next-generation immunotherapy in NSCLC. Many of these agents demonstrate encouraging response rates even in heavily pretreated populations, suggesting that novel mechanisms, particularly ADCs and bispecific constructs, may help overcome established resistance to prior immune checkpoint inhibition. Noticeably, efficacy signals appear to vary by histologic and molecular subtypes, with certain approaches (e.g., HER3- or c-MET-directed ADCs, and immune-activating bispecifics) showing greater activity in biomarker-enriched populations, underscoring the continued importance of refined patient selection beyond PD-L1 alone. In addition, toxicity profiles remain a critical consideration, particularly with cytopenias, interstitial lung disease, and immune-related adverse events, highlighting the need to balance potency with tolerability as these agents advance. Finally, the heterogeneity in outcomes across studies reinforces that no single modality is likely to be sufficient; rather, rational combination strategies and biomarker-driven approaches will be essential to fully realize the potential of these therapies.

## Figures and Tables

**Table 6:** Trials in progress without published outcomes for other classes of immunotherapies not covered separately. TAMs: tumor-associated macrophages.

Drug Class	Drug	Mechanism	NCT Number	Phase	Study Status	Combination or Monotherapy
Anchored cytokine immunotherapy	Tolododekin alfa (ANK-101)	IL-12 linked to aluminum hydroxide	NCT07027514, NCT06171750	I, I	Not yet recruiting, Recruiting	Cetrelimab, Cemiplimab
Antisense oligodeoxynucleotide (ASO)	OT-101 (Trabedersen)	TGF-β2 mRNA	NCT06579196	I/II	Recruiting	Pembrolizumab
Gene therapy	RX001 (AAV-hRUNX3)	RUNX3	NCT06934590	I	Not yet recruiting	Monotherapy
Antibody–cytokine fusion protein (Immunocytokine)	AB248	Modified IL-2 fused to anti-CD8β antibody	NCT06996782	Ib/II	Not yet recruiting	AB248, rilvegostomig, ramucirumab, chemotherapy combinations
	ANV600-001	PD-1 targeted IL-2R agonist	NCT06470763	I/II	Recruiting	Pembrolizumab
	AWT020	PD-1 nanobody with IL-2c mutein	NCT06839105	I	Recruiting	Chemotherapy, bevacizumab, and/or renvastinib combinations
	AZD6750	CD8-guided IL-2 mutein	NCT07115043	I/II	Recruiting	rilvegostomig
	IAP0971	Anti-PD-1 + IL-15/IL-15Rα complex	NCT06581419	I/II	Recruiting	Monotherapy
	PTX-912	PD-1 antibody fused to proIL-2	NCT06190886	I	Recruiting	Monotherapy
Immunomodulator	7HP349 (Alintegimod)	Activates integrins LFA-1 (αLβ2) and VLA-4 (α4β1)	NCT06362369	Ib/IIa	Recruiting	Ipilimumab, nivolumab
	BLEX 404	β-glucan polysaccharides	NCT05764928	I/II	Not yet recruiting	Pemetrexed + cisplatin
	OKN4395	Inhibits EP2, EP4, and DP1 prostanoid receptors	NCT06789172	I	Recruiting	Pembrolizumab
Oncolytic virus	VET3-TGI	Expresses IL-12, TGF-β1	NCT06444815	I	Recruiting	Pembrolizumab
	TILT-123	Expresses TNF-α and IL-2	NCT06125197	I	Recruiting	Pembrolizumab
Peptide drug conjugate	TB511	Peptide binds to CD18 on TAMs, delivering dKLA (pro-apoptotic peptide)	NCT06400160	I/IIa	Not yet recruiting	Monotherapy
Radiolabeled antibody	177Lu-RAD204	Lutetium-177 radiolabeled PD-L1 nanobody	NCT06305962	Early phase I	Recruiting	Monotherapy
	[177Lu]Lu-AKIR001	Lutetium-177 radiolabeled CD44v6 antibody	NCT06639191	Early phase I	Not yet recruiting	Monotherapy

## Data Availability

No new data were created or analyzed in this study. Data sharing is not applicable to this article.
